# Omicron Sub-Lineages (BA.1.1.529 + BA.*) Current Status in Ecuador

**DOI:** 10.3390/v14061177

**Published:** 2022-05-28

**Authors:** Andrés Carrazco-Montalvo, Andrés Herrera-Yela, Damaris Alarcón-Vallejo, Diana Gutiérrez-Pallo, Isaac Armendáriz-Castillo, Derly Andrade-Molina, Karen Muñoz-Mawyin, Juan Carlos Fernández-Cadena, Gabriel Morey-León, USFQ-COVID-19 Consortium, CRN Influenza y OVR—INSPI, Leandro Patiño

**Affiliations:** 1 Instituto Nacional de Investigación en Salud Pública “Leopoldo Izquieta Pérez”, Centro de Referencia Nacional de Genómica, Secuenciación y Bioinformática, Quito 170136, Ecuador; mherrera@inspi.gob.ec (A.H.-Y.); dalarcon@inspi.gob.ec (D.A.-V.); dgutierrez@inspi.gob.ec (D.G.-P.); 2 Instituto Nacional de Investigación en Salud Pública “Leopoldo Izquieta Pérez”, Coordinación Zonal 9, Quito 170136, Ecuador; isaac.arcas@gmail.com; 3 Facultad de Ingenierías y Ciencias Aplicadas, Universidad Internacional SEK, Quito 170302, Ecuador; 4 Omics Sciences Laboratory, Faculty of Medical Sciences, Universidad Espíritu Santo, Samborondón 092301, Ecuador; dmandrademolina@uees.edu.ec (D.A.-M.); kemunoz@espol.edu.ec (K.M.-M.); 5 INTERLAB, Guayaquil 090512, Ecuador; jfernandez@interlabsa.com; 6 Facultad de Ciencias Medicas, Universidad de Guayaquil, Guayaquil 090514, Ecuador; gabriel.moreyl@ug.edu.ec; 7 Instituto Nacional de Investigación en Salud Pública “Leopoldo Izquieta Pérez”—Dirección Técnica de Investigación, Desarrollo e Innovación, Guayaquil 170403, Ecuador

**Keywords:** COVID-19, Omicron, sub-lineages, Ecuador

## Abstract

The Omicron variant of SARS-CoV-2 is the latest pandemic lineage causing COVID-19. Despite having a vaccination rate ≥85%, Ecuador recorded a high incidence of Omicron from December 2021 to March 2022. Since Omicron emerged, it has evolved into multiple sub-lineages with distinct prevalence in different regions. In this work, we use all Omicron sequences from Ecuador available at GISAID until March 2022 and the software Nextclade and Pangolin to identify which lineages circulate in this country. We detected 12 different sub-lineages (BA.1, BA.1.1, BA.1.1.1, BA.1.1.14, BA.1.1.2, BA.1.14, BA.1.15, BA.1.16, BA.1.17, BA.1.6, BA.2, BA.2.3), which have been reported in Africa, America, Europe, and Asia, suggesting multiple introduction events. Sub-lineages BA.1 and BA.1.1 were the most prevalent. Genomic surveillance must continue to evaluate the dynamics of current sub-lineages, the early introduction of new ones and vaccine efficacy against evolving SARS-CoV-2.

## 1. Introduction

The novel coronavirus identified as severe acute respiratory syndrome coronavirus 2 (SARS-CoV-2) is the infectious agent causing the COVID-19 pandemic [1]; by April 2022, a total of 483,556,595 positive cases and 6,132,461 deaths worldwide had been reported [2]. The mutation rate of the virus is still in debate, but after two years, it has evolved into different variants, some of which have higher transmission rates [3]. Some model studies suggest that the effective (instantaneous) reproduction number of Omicron could be from 3.19 (95% CI: 2.82–3.61) [4,5] to 3.3 (95% CI: 2.0–7.8) [6], which is greater than that of the Delta variant. Moreover, each emerging variant could diverge into sub-lineages that need to be surveilled locally in order to evaluate any change in their transmission dynamics.

Omicron, unlike previous variants, harbors a wide variety of mutations within its genome [7]. Fifteen mutations have been reported only in the Receptor Binding Domain (RBD) region, which enable Omicron to be more transmissible by allowing the virus to bind more easily to the human angiotensin-converting enzyme 2 (ACE2) protein as compared with the original strain [8]. Other relevant mutations include R203K and G204R, which are linked to viral replication [4]. According to WHO, by April 2022 [9], five major sub-lineages originating from BA.1.1.529 had been detected globally: BA.1, BA.2, BA.3, BA.4, BA.5 with different frequencies in different regions.

Ecuador is among the countries with a vaccine coverage ≥85%; however, following the introduction of the Omicron Variant (lineage BA.1.1.529 in December 2021) [10] the Ministry of Public Health reported the highest frequency of positive cases since the beginning of the pandemic, accumulating more than 300,000 cases from January to March 2022 [11]. By the end of March, sub-lineage BA.2 was also detected.

At least four institutions from the public and private sector are involved locally in the genomic surveillance of the virus and are submitting sequences to the public repository GISAID with a two-week frequency [12]. This allows us to understand the epidemiological trends and incidence of the COVID-19 disease and the effects of the detection and spread of new variants [13]. Given the emergence of sub-lineages, this work aims to identify which are circulating and prevailing in Ecuador. Our analyses focused on the presence and trend of each Omicron sub-lineage (BA.1.1.529 + BA.*) reported since epidemiological week 49 in 2021, when we detected the first case in Ecuador [10].

## 2. Materials and Methods

### 2.1. Sequence Production and Data Collection

The institutions involved in SARS-CoV-2 sequencing in Ecuador use different platforms including MinION (Oxford Nanopore), MiSeq and MiniSeq (Illumina). We downloaded 1245 Omicron sequences submitted to GISAID from Ecuador up until March 2022; 703 obtained from “Instituto Nacional de Investigación en Salud Publica (INSPI)” using MinION (ONT) and MiSeq (Illumina), 456 obtained from “Universidad San Francisco de Quito-USFQ” using MinION and 86 sequences obtained from “Universidad de Especialidades Espiritu Santo” which used MiniSeq (Illumina). Starting from the date on which the first case of Omicron was detected in Ecuador, a comparison of sequences was performed by epidemiological week for Omicron vs. Delta.

### 2.2. Lineage Assignment and Phylogenetics

Sequences were classified by epidemiological week and then submitted to Nextclade [14] and Pangolin COVID-19 [15] for clade and lineage assignment. Lineage nomenclature was assigned according to the PANGO (Phylogenetic Assignment of Named Global Outbreak Lineages) software updated in March 2022. It relies on establishing a numerical value to descendants that meet certain conditions that belong to lineages A or B, with a maximum of three sublevels, whereby new lineages will be assigned with a letter [15]. The criteria used for lineage assignment involved minimum lineage size, genome quality, genetic specificity, and epidemiological significance, which vary over time and depend on the degree of adaptation [16]. Consequently, each lineage is assigned a unique alphanumeric code that includes partial information regarding the phylogenetic history of that lineage based on a common ancestor [15].

A stacked bar chart of lineages by epidemiological week was made using R [17] and GraphPad Prism software [18]. A phylogenetic tree was built in Nextclade using the nearest neighbor method and visualized by Nextrain Auspice [14].

## 3. Results

**Figure 1:**
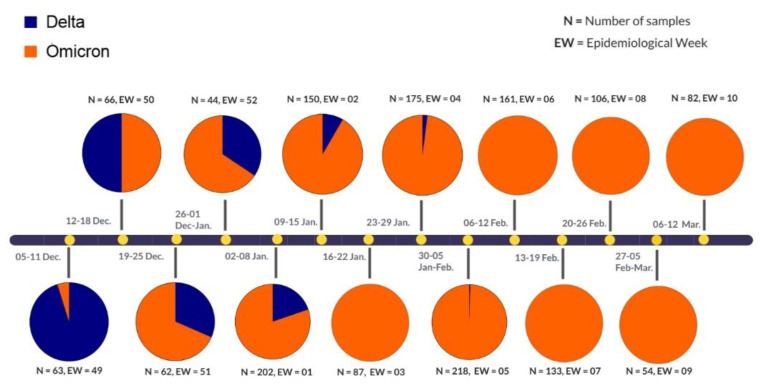
Variants of concern in Ecuador since Omicron’s first detection.

The first case of Omicron was detected in Ecuador in epidemiological week (EW) 49 of 2021, it co-circulated with Delta until EW 52 of 2021 and EW 05 of 2022. From EW 06 Omicron became the only variant detected in all the samples sequenced, Figure 1.

We detected 12 sub-lineages of the Omicron variant (BA.1.1.529 + BA.*) circulating in Ecuador, with different worldwide origins and mutation numbers (Table 1). All sub-lineages display the highest number of mutations in the spike protein. BA.2 and BA.2.3 sub-lineages differ from the others in the number of mutations in five out of nine genes (ORF1a, ORF1b, S, M, N). The complete set of mutations is detailed in Table S1 [15].

**Figure 2:**
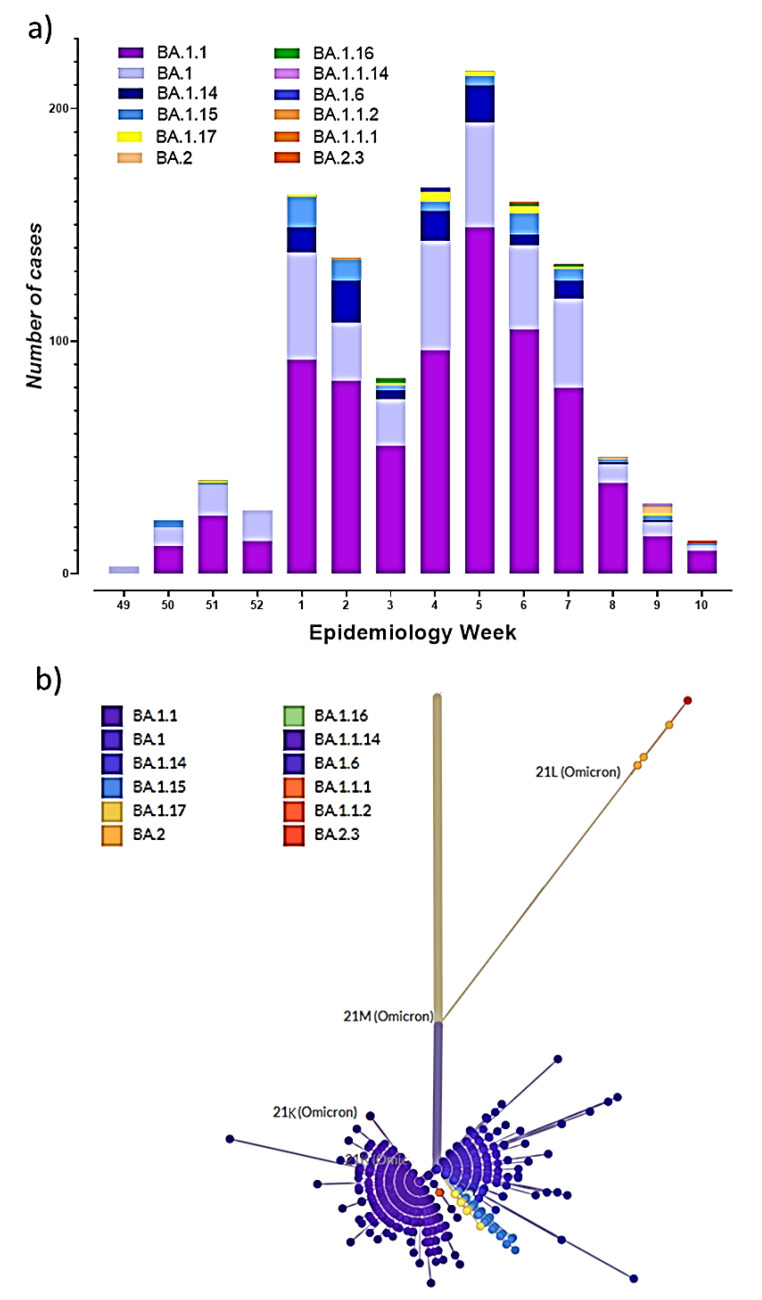
Omicron sub-lineages (BA.1.1.529 + BA.*) in Ecuador. (**a**) Sub-lineages by epidemiological week, (**b**) phylogenetic tree of all lineages.

Figure 2a shows the number of cases for each sub-lineage from EW 49 of 2021 to EW 10 of 2022. BA.1 was the first sub-lineage detected followed by BA.1.1 and BA.1.15 which were detected on EW 50 of 2021. BA.1.16 and BA.1.17 appeared on EW 51 of 2021, all remaining sub-lineages have been detected since EW 2 of 2022. BA.1 and BA.1.1 were found across all epidemiological weeks, while the growth and diversity of the other sub-lineages were proportional with the increase of cases; however, both decreased together when positive cases dropped (Table S2). Among all the sequences analyzed, 62.33% corresponded to the BA.1.1 sub-lineage, 24.82% to BA.1, 6.18% to BA.1.14, 4.33% to BA.1.15, 1.12% to BA.1.17, the remaining sub-lineages BA.1.6, BA.1.16, BA.2, BA.2.3, BA.1.1.1; BA.1.1.2, BA.1.1.14, were found with a frequency ≤1%.

The phylogenetic analysis of the sub-lineages detected is shown in Figure 2b. The sub-lineages clustered into two clades: 21K and 21L, which diverged from 21M. The highest diversity was in the 21K clade, forming two groups with BA.1 and BA.1.1. The 21K clade only showed two sub-lineages corresponding to BA.2 and BA.2.3.

Among the most predominant sub-lineages registered in Ecuador, BA.1 appeared in 162 countries showing higher prevalence in the UK at 43.0%, USA 22.0%, Denmark 5.0%, Germany 4.0%, and Brazil 3.0%; BA.1.1 appeared in 154 countries showing higher prevalence in the USA at 48.0%, UK 22.0%, Germany 6.0%, Canada 4.0%, and France 2.0%; BA.1.14 appeared in 82 countries with higher prevalence in Brazil at 19.0%, Belgium 17.0%, UK 13.0%, Denmark 10.0% and Germany 7.0%; and BA.1.15 appeared in 124 countries with most prevalence in the USA at 69.0%, the UK 14.0%, Canada 4.0%, Germany 2.0% and Mexico 1.0% [15].

The BA.2 and BA.2.3 sub-lineages were recently reported in Ecuador; these currently predominate in several countries, so we analyzed their worldwide prevalence: BA.2 appeared in 119 countries with higher prevalence in the UK at 40.0%, Denmark 16.0%, Germany 13.0%, the USA 7.0% and France 4.0% [15]; and BA.2.3 has been registered in 81 countries with higher prevalence in the UK at 31.0%, the USA 23.0%, Canada 10.0%, Germany 4.0%, and South Korea 4.0% [15].

## 4. Discussion

Following Omicron ߣs first identification, an increasing number of sub-lineages are being reported globally. Of all the worldwide Omicron sequences available in GISAD, at least 36 sub-lineages (all in the major BA.1–BA.5 as reported by WHO) had been identified by April 2022 [9,19]. In Ecuador, up to March 2022, we had detected 12 Omicron sub-lineages (BA.1.1.529 + BA.*) also reported in Africa, America, Europe, and Asia, suggesting multiple introduction events. The detected sub-lineages harbor from 40 to 54 mutations (Table 1), of which approximately 30 contribute to amino acid changes in the SARS-CoV-2 spike protein [20].

During the Omicron wave in Ecuador, the major number of cases occurred between epidemiological weeks one and seven of 2022 (Figure 2a). However, in EW04 and EW05, the Delta variant was still circulating in less populated provinces in the country. At the end of December 2021, sub-lineages BA.1 and BA.1.1 were the most predominant, which agreed with the global trends [21]. In Europe, Denmark had reported the prevalence of BA.1, BA.1.1, and BA.2 sub-lineages in the same period [22]. In Italy, the predominant sub-lineages were BA.1.15, BA.1.1, and BA.1.17 [19]. A study conducted in Hong Kong using 542 Omicron sequences, showed the BA.2.2, BA.1 and BA.1.1 sub-lineages predominated [23]. According to PAHO reports, BA.1 and BA.1.1 were also identified in more than 97% of the cases registered in the Americas [24]. Different Omicron sub-lineages (BA.1.1.529 + BA.*) have also been reported in other Latin American countries; a study by the University of Feevale in Brazil has reported the circulation of seven sub-lineages in that country, with BA.1, BA.1.1 and lately BA.2 being the predominant ones [25]. A similar pattern occurred in Chile, where the Ministry of Health reported the circulation of 10 sub-lineages, with BA.1.1 and BA.2 displacing BA.1 [26].

Our analysis shows the divergence of the 21K and 21L clades from 21M, which is related to three mutations in 21L not present in 21K: they comprise Nsp3 (G489S, analogous to the A488S mutation found in Delta VOC), Nsp4 (L438F, analogous to the L438P mutation found in Lambda VOC) and Nsp6 which displaced the C-terminus in the 21K clade [27]. Differences in the number of N mutations were also identified, the BA.2 and BA.2.3 sub-lineages showed an R203K/G204R mutation in the nucleocapsid protein. This mutation has also been reported by Wu, H., et al. (2021), being associated with the appearance of new variants [28].

In this study, five samples were identified within Clade 21L (BA.2 and BA.2.3 lineages) in EW 10; due to the high transmissibility and predominance of BA.2 and BA.2.3 reported in other countries, it is important to survey them in Ecuador during the following months. According to the United Kingdom Health Security Agency, the BA.2 sub-lineage has shown a higher growth rate than BA.1; in England, BA.2 showed 75% more spreading when compared to BA.1 [29]. Similar behavior was observed in Denmark, where BA.2 had an accelerated growth compared to BA.1, becoming the dominant sub-lineage [30]. The behavior of BA.2 may be related to mutations found in the spike protein of the virus [30]. According to the detail of the mutations presented in the results section of this study, one of the main differences between BA.1 and BA.2 is the absence of the 143/145 deletion in BA.2, as mentioned by Colson et al.; this difference produces a flattening of the surface of the N-terminal domain (NTD) that could facilitate the initial interaction of the virus with the lipid rafts and would explain its greater transmissibility [31]. Another difference is that BA.1 and BA.3 sub-lineages have a deletion 69/70 in the spike protein, which is not found in BA.2 [31].

BA.2 sub-lineage was detected in Ecuador in EW 10, coinciding with a drop in case numbers, probably due to a high incidence of the first Omicron sub-lineage (BA.1.1.529 + BA.*) combined with the high vaccination coverage. The COVID-19 positivity from EW 19 was ≤5% [32]. Despite a low positivity rate detected in the last weeks, Public Health Authorities need to keep all the genomic surveillance strategies active to evaluate any change in the predominance of the Omicron sub-lineage (BA.1.1.529 + BA.*) currently circulating in the country. Furthermore, early detection of new lineages reported worldwide will help to improve current public health policies and influence decisions such as not requiring people to wear a mask in outdoor areas of conveyances. Furthermore, these reports will provide information on the efficacy of the vaccines as well as the future reinforcement doses required to protect the most vulnerable population from the emergence of new variants and sub-lineages.

The current pandemic has highlighted the constant danger of the emergence or re-emergence of viruses with pandemic potential. SARS-CoV-2 has demonstrated a high capacity for evolution and adaptability; it has mutated at an accelerated rate, leading to new variants [33]. Therefore, the response to the possible emergence of new viruses involves scientific and technological strengthening of research to consolidate a surveillance system with a preventive approach for the containment of future outbreaks [34].

## Figures and Tables

**Table 1:** List of Omicron sub-lineages circulating in Ecuador. For each Omicron sub-lineage, the place/s of origin and mutation numbers that characterize them are recorded.

Sub-Lineage	Clade Names	Origin	Genes								
			ORF1a	ORF1b	S	ORF3a	E	M	ORF6	ORF8	N
BA.1	21K (Omicron)	South Africa	8	2	33	0	1	3	0	1	4
BA.1.1	21K (Omicron)	South Africa	8	2	32	0	1	3	0	1	4
BA.1.1.1	21K (Omicron)	Europe	8	2	31	0	1	3	0	1	4
BA.1.1.14	21K (Omicron)	Europe	8	2	30	0	1	3	0	1	4
BA.1.1.2	21K (Omicron)	Japan	9	2	34	0	1	3	0	1	4
BA.1.14	21K (Omicron)	Brazil	8	2	21	0	1	3	0	1	4
BA.1.15	21K (Omicron)	USA	8	2	30	1	1	3	0	1	5
BA.1.16	21K (Omicron)	UK	8	2	29	0	1	3	0	1	4
BA.1.17	21K (Omicron)	Europe	9	2	30	0	1	3	0	1	4
BA.1.6	21K (Omicron)	Canada and Sint Maarten	8	3	30	0	1	3	0	1	4
BA.2	21L (Omicron)	India and South Africa	9	4	29	1	1	2	1	1	5
BA.2.3	21L (Omicron)	Philippines	10	4	29	2	1	2	1	1	5

## Data Availability

Not applicable.

## Supplementary Materials

The following supporting information can be downloaded at: https://www.mdpi.com/article/10.3390/v14061177/s1, Table S1: Detail of mutations by sub-lineage, Table S2: Number of cases by epidemiological week.
